# Clinical Frailty Scale, Surprise Question and 1-year Mortality in Older Patients with Advanced CKD

**DOI:** 10.34067/KID.0000000936

**Published:** 2025-08-21

**Authors:** Imre Demirhan, Micha Jongejan, Mathijs van Oevelen, Keanu Kiriwenno, Simon P. Mooijaart, Marianne C. Verhaar, Willem Jan W. Bos, Hanneke Joosten, Trijntje T. Cnossen, Marjolijn van Buren, Alferso C. Abrahams

**Affiliations:** 1 Department of Nephrology and Hypertension, University Medical Center Utrecht, Utrecht, The Netherlands; 2 Department of Internal Medicine, Leiden University Medical Center, Leiden, The Netherlands; 3 Department of Internal Medicine, Section of Gerontology and Geriatrics, Leiden University Medical Center, Leiden, The Netherlands; 4 LUMC Center for Medicine for Older People, Leiden University Medical Center, Leiden, The Netherlands; 5 Department of Internal Medicine, St. Antonius Hospital, Nieuwegein, The Netherlands; 6 Division of General Internal Medicine, Department of Internal Medicine, Section Geriatric Medicine, Maastricht University Medical Center+, Maastricht, The Netherlands; 7 Department of Internal Medicine, Amphia Hospital, Breda, The Netherlands; 8 Department of Internal Medicine, Haga Hospital, The Hague, The Netherlands

**Keywords:** CKD, clinical nephrology, geriatric nephrology, mortality, mortality risk, risk factors, survival, longitudinal data analysis, survival analysis

## Abstract

**Key Points:**

- Both Clinical Frailty Scale score ≥5 and Surprise Question answer no are associated with higher 1-year mortality risk in older patients with advanced CKD.
- The strongest association with mortality can be found by combining assessments when both indicate high risk.
- Implementing the Clinical Frailty Scale and Surprise Question may help guide decisions for either KRT or conservative kidney management and highlight patients for whom advance care planning should be initiated.

**Background:**

Frailty is common in older patients with advanced CKD and is associated with mortality. This study investigates whether the Clinical Frailty Scale (CFS) and Surprise Question (Would you be surprised if this patient died in the next 12 months?, SQ) are associated with 1-year mortality and whether combining risk assessments has benefits.

**Methods:**

Patients ≥65 years with eGFR 20–10 ml/min per 1.73 m^2^ were included from the ongoing prospective observational cohort study DIALysis or not: Outcomes in older kidney patients with GerIatriC Assessment (first inclusion May 13th, 2020). Frailty was screened using the CFS, and the SQ was answered using clinical impression (gestalt). Patients were classified high risk with CFS score ≥5 and/or SQ answer no. Four subgroups were formed: high risk, CFS ≥5 & SQ no; high risk, CFS ≥5 only; high risk, SQ no only; and low risk, CFS <5 & SQ yes. Associations with 1-year mortality were explored using Kaplan–Meier curves and adjusted Cox proportional hazards models.

**Results:**

Overall, 589 patients were included (male sex 70%, mean age 77±6 years, mean eGFR 15±3 ml/min per 1.73 m^2^). CFS score ≥5 was found in 125 patients (21%) and 112 patients (19%) had SQ answer no. Both CFS score ≥5 (adjusted hazard ratio [HR], 3.09; 95% CI, 1.75 to 5.54) and SQ answer no (adjusted HR, 1.96; 95% CI, 1.09 to 3.52) were associated with higher mortality risk. Subgroup high risk, CFS ≥5 and SQ no had the highest mortality risk (adjusted HR, 3.37; 95% CI, 1.65 to 6.91).

**Conclusions:**

Both CFS score ≥5 and SQ answer no are associated with higher 1-year mortality risk in older patients with advanced CKD. The strongest association with mortality was found by combining both assessments, when both indicate high risk. These findings may help older patients and nephrologists make better informed treatment decisions and initiate timely advance care planning conversations.

## Visual Abstract

**Figure:**
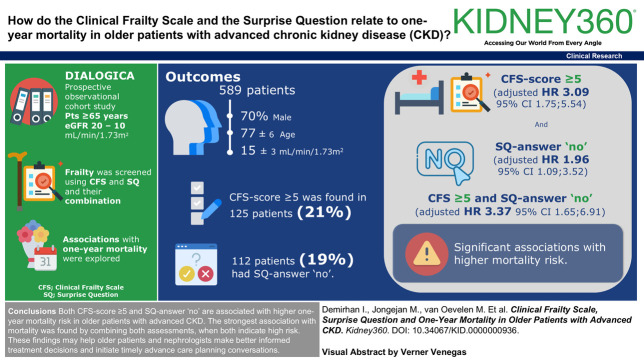

## Introduction

CKD is highly prevalent in older patients, affecting up to 39% of patients age ≥60 years.^1^ CKD-linked processes (*e.g*., inflammation, toxin accumulation, low energy intake, metabolic/hormonal dysregulation) contribute to frailty in this population.^2,3^ Frailty is marked by low physical activity, reduced strength, multiorgan deficits, reduced reserves, and vulnerability to stressors, and is associated with high mortality and impaired health-related quality of life (HRQoL) in patients with CKD.^4–6^ Frailty assessment could therefore aid in shared decision making (SDM) regarding KRT or conservative kidney management (CKM), as KRT may be unsuitable for some patients because of potential high treatment burden and early mortality.^7,8^ Furthermore, frailty assessment may identify patients who benefit from advance care planning (ACP) initiation.^9^

Many extensive frailty assessment tools exist for CKD populations with varying strengths and limitations, typically requiring geriatric referral.^3^ The Clinical Frailty Scale (CFS) instead is an easily applicable screening tool evaluating somatic, cognitive, and functional characteristics and can be implemented by nephrology care providers.^10,11^ Another quick tool is the Surprise Question (Would you be surprised if this patient died in the next 12 months?, SQ), also implementable by nephrology care providers.^12,13^ In contrast to the CFS, the SQ relies on the subjective clinician's impression and intuition (gestalt) of a patient's health status and prognosis to estimate mortality risk.^14^ Both the CFS and SQ are part of a consensus-based nephrology-tailored geriatric assessment tailored for older patients with CKD to evaluate somatic status, helping identify patients at high risk and thus guide treatment decisions.^15^

It remains unclear whether frailty and risk assessment using the CFS and SQ are associated with early mortality, specifically in older patients with advanced CKD approaching kidney failure, and whether combining both is associated with higher mortality risk. Exploring this may provide important information to guide SDM for nephrologists and their patients. Thus, this study aims to investigate the association between both frailty and clinical impression using the CFS and SQ with 1-year mortality in older patients with advanced CKD. Furthermore, this study explores whether combining both assessments is of added value compared with either assessment individually.

## Methods

### Study Design and Participants

This study included patients from the DIALysis or not: Outcomes in older kidney patients with GerIatriC Assessment (DIALOGICA) study, which is an ongoing multicenter prospective observational study in 40 hospitals in the Netherlands (38 hospitals) and Belgium (2 hospitals). DIALOGICA aims to compare outcomes (including HRQoL, clinical outcomes and costs) of older patients with kidney failure opting for either KRT or CKM. Patients are eligible for inclusion if age ≥65 years, Dutch speaking, and with an eGFR between 20 and 10 ml/min per 1.73 m^2^, determined with the CKD Epidemiology Collaboration 2009 equation, or an average of urea and creatinine clearance in 24-hour urine between 20 and 10 ml/min. Participants provided informed consent before study participation. Data are collected yearly after inclusion when eGFR is between 20 and 10 ml/min per 1.73 m^2^ and yearly after either eGFR drops to ≤10 ml/min per 1.73 m^2^ or KRT is initiated, until patient death or loss to follow-up. This includes data from nephrology-tailored geriatric assessments, providing information on geriatric domains involving assessments of the CFS and SQ as well.^15^ Clinical and demographical information are derived from questionnaires and electronic patient records. A more detailed description of the methodology of the DIALOGICA study can be found in the rationale paper.^16^ The study is conducted according to the principles of the Declaration of Helsinki and the International Council for Harmonisation - Good Clinical Practice guidelines and approved by the Medical Ethics Review Committee South-West Holland (METC Zuidwest Holland, reference number 19-071). The first patient was included on May 13th, 2020. For this study, all DIALOGICA participants who were included from May 13th, 2020, up to April 15th, 2024, who had data available on both the CFS and SQ assessments at baseline were included. The results are reported in accordance with the STrengthening the Reporting of OBservational studies in Epidemiology guidelines.^17^ The filled in STrengthening the Reporting of OBservational studies in Epidemiology checklist is included as Supplemental Material.

### Measurements

#### CFS and SQ

In DIALOGICA, both the CFS (version 2.0) and SQ were assessed as part of nephrology-tailored geriatric assessments, performed by doctors (geriatricians, internists, nephrologists), a nurse, or a health care professional trained to perform this assessment.^15^ The CFS is a quick tool to assess frailty which includes evaluations of different patient characteristics, such as cognitive functioning, functional status, and severity of comorbidities.^11,18^ In the CFS version 2.0 has scores that range from 1 (very fit) to 9 (terminally ill) and provides visual drawings and brief descriptions per score to help guide scoring by the user.^11,19^ It is both easy to use and can be completed quickly.^20^ It has been validated for use in populations with advanced CKD.^3^ CFS scores of ≥5 and <5 were used to group patients as frail (thus high risk) and nonfrail (low risk), respectively, in line with the original and most frequently used cutoff point.^18,21^

The SQ (Would you be surprised if this patient died in the next 12 months?) was answered after the CFS, resulting in two possible answers: no (*I would not be surprised*) or yes (*I would be surprised*). The SQ relies on a clinician's subjective impression or gestalt of the patient's health status and expected prognosis to identify patients with high mortality risk and is meant to prompt care providers to consider ACP conversations.^14,22^ Patients with no answers were grouped as high risk, and patients with yes answers as low risk. The use of the SQ is validated for use in many different populations, including patients with advanced CKD.^13,23^

Patients were categorized into four risk subgroups based on the results of the CFS and SQ assessments: high risk, CFS ≥5 & SQ no; high risk, CFS ≥5 only; high risk, SQ no only; and low risk, CFS <5 & SQ yes (used as reference group for subgroup analyses).

#### Outcome Assessment

The primary outcome of interest is time to all-cause death, with follow-up starting from the date of inclusion and restricted at 1 year. The time to event was obtained from electronic patient files in the participating hospitals or by consulting primary care facilities. Patient survival was censored when consent was withdrawn by the patient, in the case of patient transfer to a different nephrology care facility not participating in DIALOGICA (loss to follow-up), or in the case of follow-up time <1 year (patients included <12 months before April 15th, 2024).

### Statistical Analyses

Frequencies and percentages for categorical data and means with SD or medians with interquartile range (IQR), dependent on data distribution, were calculated for the baseline characteristics. This was done for the entire cohort and stratified for the results of the CFS (score ≥5, score <5), SQ (answer no, yes), and for the four aforementioned risk subgroups. To explore potential selection bias, differences in baseline characteristics between included and excluded patients (lacking CFS and/or SQ assessment) were compared as well.

To assess the association between the results of the CFS and SQ assessments and 1-year all-cause mortality, Kaplan–Meier mortality curves (including log-rank tests) and Cox proportional hazards model analyses were performed using the CFS score (score ≥5 versus <5) and SQ answers (no versus yes). The Cox proportional hazards models were adjusted for potential confounders, which were added in steps. Model 1 comprised only either the CFS or SQ assessment (crude), model 2 added age and sex (partially adjusted), and model 3 finally added eGFR (fully adjusted).

To assess whether combining the results of both the CFS and SQ is of added value for mortality risk estimation, associations between the four subgroups and 1-year mortality were explored as well using the same steps, using the subgroup low risk, CFS <5 & SQ yes as the reference group. Residual plots and time-dependent interaction terms were examined for all Cox proportional hazards models to confirm that the proportional hazard assumption was not violated. To explore whether selection bias may have affected our analyses, the 1-year mortality risk of included and excluded patients (lacking CFS and/or SQ assessment) were compared as well by using a Cox proportional hazards model.

A *P* value of <0.05 was considered statistically significant for all the aforementioned analyses. None of the covariates used in the multivariable models had missing values. All analyses were performed in SPSS version 30.0.0.

## Results

### Baseline Characteristics

**Figure 1:**
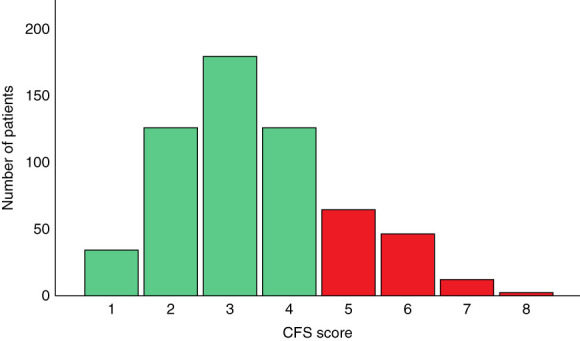
**Histogram showing the distribution of CFS score of the cohort at baseline.** Histogram showing number of patients per CFS score, with green indicating nonfrail (score CFS score <4) and red indicating frail (CFS score ≥5). CFS score 1 (very fit) *n*=34. CFS score 2 (fit) *n*=126. CFS score 3 (managing well) *n*=180. CFS score 4 (living with very mild frailty) *n*=124. CFS score 5 (living with mild frailty) *n*=65. CFS score 6 (living with moderate frailty) *n*=46. CFS score 7 (living with severe frailty) *n*=12. CFS score 8 (living with very severe frailty) *n*=2. CFS score 9 (terminally ill) *n*=0. CFS, Clinical Frailty Scale.

On April 15th, 2024, 711 patients were included in the DIALOGICA study. In total, 589 patients of these patients (83%) had both CFS and SQ assessments available and were thus included in this study (displayed in Supplemental Figure 1). The median time from inclusion to assessment of the CFS and SQ was 21 days. Baseline patient characteristics are displayed in Table 1. Most patients were male (70%), the mean age was 77 (SD 6) years, and the mean eGFR was 15 ml/min per 1.73 m^2^ (SD 3). The distribution of CFS scores of the cohort is shown in Figure 1, with a median score of 3 (IQR, 2–4). In total, 125 patients (21%) had CFS scores ≥5 and 112 patients (19%) had SQ answer no. Patients with CFS score ≥5 were older, had more symptoms, used more medications, and had worse nutritional statuses than patients with CFS score <5. This was also the case for patients with SQ answer no compared with SQ answer yes.

**Table 1:** Baseline patient characteristics of the entire cohort and stratified for Clinical Frailty Scale and Surprise Question

Patient Characteristics	Entire Cohort (*N*=589)	CFS		SQ	
		Score ≥5 (*n*=125)	Score <5 (*n*=464)	Answer No (*n*=112)	Answer Yes (*n*=477)
Age in yr, mean (SD)	77 (6)	78 (6)	76 (6)	79 (6)	76 (6)
Male sex, *No.* (%)	410 (70)	71 (57)	339 (73)	80 (71)	330 (69)
eGFR in ml/min per 1.73 m^2^, mean (SD)	15 (3)	15 (3)	15 (3)	15 (3)	15 (3)
**Primary kidney disease, *No.* (%)**					
Diabetic kidney disease	123 (21)	24 (20)	99 (22)	25 (22)	98 (21)
GN	30 (5)	4 (3)	26 (6)	5 (5)	25 (5)
Hypertension	107 (19)	21 (17)	86 (19)	20 (18)	87 (19)
Cystic kidney disease	25 (4)	5 (4)	20 (4)	3 (3)	22 (5)
Pyelonephritis	4 (1)	1 (1)	3 (1)	0 (0)	4 (1)
Renal vascular disease	105 (18)	26 (22)	79 (17)	19 (17)	86 (18)
Other	149 (26)	30 (24)	119 (26)	31 (28)	118 (25)
Unknown	34 (6)	10 (8)	24 (5)	8 (7)	26 (6)
**Living situation, *No.* (%)**					
Alone, independently	200 (38)	40 (39)	160 (38)	32 (33)	168 (39)
With partner	302 (58)	54 (52)	248 (59)	57 (59)	245 (58)
With family	17 (3)	6 (6)	11 (3)	5 (5)	12 (3)
Nursing home	4 (1)	4 (4)	0 (0)	3 (3)	1 (0)
**Educational level, *No.* (%)** ^a^					
Low	198 (37)	47 (42)	166 (36)	39 (38)	159 (36)
Medium	181 (34)	37 (33)	157 (34)	32 (31)	149 (34)
High	161 (30)	28 (25)	141 (31)	32 (31)	129 (30)
ADL–Katz score, median (IQR)^b^	0 (0–1)	1 (0–2)	0 (0–1)	1 (0–2)	0 (0–1)
IADL–Lawton score, median (IQR)^b^	5 (5–5)	5 (3–6)	5 (5–5)	5 (3–5)	5 (5–5)
No. of DSI symptoms, median (IQR)	10 (6–14)	14 (9–18)	9 (6–13)	12 (8–17)	10 (6–13)
Charlson Comorbidity Index score, median (IQR)	4 (3–5)	4 (3–5)	4 (3–5)	4 (3–6)	4 (3–5)
No. of medications, median (IQR)	11 (8–14)	12 (10–16)	10 (7–13)	12 (9–14)	10 (7–14)
**Mini nutritional assessment status, *N* (%)**					
Well nourished	429 (74)	76 (63)	353 (77)	62 (57)	367 (78)
At risk of malnutrition	136 (23)	40 (33)	96 (21)	39 (36)	97 (21)
Malnourished	15 (3)	5 (4)	10 (2)	7 (7)	8 (2)
Serum albumin in g/L, mean (SD)	38.3 (5.3)	37.9 (6.1)	38.5 (5.1)	37.9 (6.4)	38.4 (5.1)
**Smoking status, *No.* (%)**					
Quit	346 (63)	72 (65)	274 (63)	69 (67)	277 (63)
Current smoker	46 (8)	6 (5)	40 (9)	7 (7)	39 (9)
**Alcohol use, *No.* (%)**					
Never or previously quit	310 (58)	75 (70)	235 (55)	69 (69)	241 (55)
Current user	228 (42)	32 (30)	196 (46)	31 (31)	197 (45)

ADL, Activities Of Daily Living; CFS, Clinical Frailty Scale, score of ≥5 classified as high risk and <5 as low risk; DSI, Dialysis Symptom Index; IADL, Instrumental Activities Of Daily Living; IQR, interquartile range; SQ, Surprise Question, no classified as high risk and yes classified as low risk.

a Based on the Dutch Verhage education classification.

b Activities of daily living, basic and essential self-care tasks. Lower ADL–Katz score reflects more independence, range 0–12. Instrumental activities of daily living, more complex activities of daily living tasks. Higher Instrumental activities of daily living–Lawton score reflects more independence, range 0–5 in men and 0–8 in women. Missing values: serum albumin 14%, primary kidney disease 2%, living situation 11%, educational level 8%, ADL–Katz score 13%, instrumental activities of daily living-Lawton score 12%, number of dialysis symptom index symptoms 6%, number of medications 2%, mini nutritional assessment 2%, smoking status 7%, alcohol use 9%.

Baseline characteristics for the four subgroups are displayed in Table 2. Patients in subgroups high risk, CFS ≥5 & SQ no; high risk, CFS ≥5 only; and high risk, SQ no only were older, had more symptoms, had higher Charlson Comorbidity Index scores, used more medications, and had worse nutritional statuses, compared with the reference subgroup low risk, CFS <5 & SQ yes.

**Table 2:** Patient characteristics of the four screening tool assessment subgroups

Patient Characteristics	High risk, CFS ≥5 & SQ No (*n*=63)	High risk, CFS ≥5 Only (*n*=62)	High risk, SQ No Only (*n*=49)	Low risk, CFS <5 & SQ Yes (*n*=415)
Age in yr, mean (SD)	80 (6)	77 (6)	79 (5)	76 (5)
Male sex, *No.* (%)	39 (62)	32 (52)	41 (84)	298 (72)
eGFR in ml/min per 1.73 m^2^, mean (SD)	15 (3)	15 (3)	14 (3)	15 (3)
**Primary kidney disease, *No.* (%)**				
Diabetic kidney disease	12 (19)	12 (20)	13 (27)	86 (21)
GN	2 (3)	2 (3)	3 (6)	23 (6)
Hypertension	12 (19)	9 (15)	8 (16)	78 (19)
Cystic kidney disease	2 (3)	3 (5)	1 (2)	19 (5)
Pyelonephritis	0 (0)	1 (2)	0 (0)	3 (1)
Renal vascular disease	10 (16)	16 (27)	9 (18)	70 (17)
Other	19 (31)	11 (19)	12 (25)	107 (26)
Unknown	5 (8)	5 (9)	3 (6)	21 (5)
**Living situation, *No.* (%)**				
Alone, independently	17 (32)	23 (46)	15 (35)	145 (39)
With partner	30 (56)	24 (48)	27 (63)	221 (59)
With family	4 (7)	2 (4)	1 (2)	10 (3)
Nursing home	3 (6)	1 (2)	0 (0)	0 (0)
**Educational level, *No.* (%)** ^a^				
Low	26 (46)	21 (38)	13 (28)	138 (36)
Medium	16 (28)	21 (38)	16 (35)	128 (34)
High	15 (26)	13 (24)	17 (37)	116 (30)
ADL-Katz score, median (IQR)^b^	1 (0–2)	1 (0–2)	0 (0–1)	0 (0–1)
IADL–Lawton score, median (IQR)^b^	5 (3–6)	5 (3–6)	5 (4–5)	5 (5–5)
No. of DSI symptoms, median (IQR)	15 (10–18)	12 (8–18)	10 (7–14)	9 (6–13)
Charlson Comorbidity Index score, median (IQR)	4 (3–5)	5 (4–5)	5 (3–6)	3 (3–5)
No. of medications, median (IQR)	13 (9–16)	13 (10–16)	11 (8–13)	10 (7–13)
**Mini nutritional assessment status, *No.* (%)**				
Well nourished	32 (53)	44 (72)	30 (63)	323 (79)
At risk of malnutrition	25 (42)	15 (25)	14 (29)	82 (20)
Malnourished	3 (5)	2 (3)	4 (8)	6 (2)
Serum albumin in g/L, mean (SD)	37.3 (6.5)	37.9 (5.5)	37.7 (6.6)	38.6 (4.8)
**Smoking status, *No.* (%)**				
Quit	36 (62)	36 (68)	33 (73)	241 (62)
Current smoker	3 (5)	3 (6)	4 (9)	36 (9)
**Alcohol use, *No.* (%)**				
Never or previously quit	40 (73)	35 (67)	29 (65)	206 (53)
Current user	15 (27)	17 (33)	16 (36)	180 (47)

ADL, Activities Of Daily Living; CFS, Clinical Frailty Scale, score of ≥5 classified as high risk and <5 as low risk; DSI, Dialysis Symptom Index; IADL, Instrumental Activities Of Daily Living; IQR, interquartile range; SQ, Surprise Question, no classified as high risk and yes classified as low risk.

a Based on the Dutch Verhage education classification.

b Activities of daily living, basic and essential self-care tasks. Lower ADL–Katz score reflects more independence, range 0–12. Instrumental activities of daily living, more complex activities of daily living tasks. Higher instrumental activities of daily living–Lawton score reflects more independence, range 0–5 in men and 0–8 in women.

Supplemental Table 1 shows the differences in baseline characteristics between included and excluded patients. Excluded patients were older, had lower education levels, had worse activities of daily living (ADL) scores, and had more symptoms. Their mortality rate was significantly higher than the included patients (17% versus 9%, *P* < 0.001).

### CFS, SQ, and 1-Year Mortality

**Figure 2:**
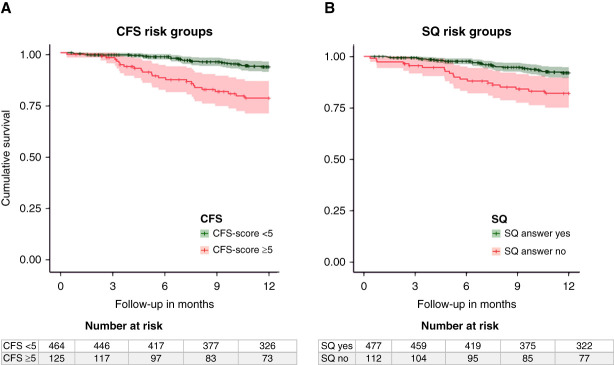
**Kaplan–Meier curves of CFS (left image) and SQ (right image) and 1-year all-cause mortality.** Group with CFS score ≥5: 19% mortality rate after 1 year versus group with CFS score <5: 6% mortality rate after 1 year (log-rank *P* value <0.001). Group with SQ answer no: 17% mortality rate after 1 year versus group with SQ answer yes: 7% mortality rate after 1 year (log-rank *P*-value <0.001). SQ, Surprise Question.

The Kaplan–Meier curves depicting 1-year mortality for both the CFS and SQ assessment results are displayed in Figure 2, A and B. The median follow-up time was 12 months (IQR, 10–12). Of the 589 patients at baseline, 15 (3%) were lost to follow-up, and 52 patients (9%) had died within 1 year. In total, 24 of 125 (19%) patients with CFS score ≥5 died, compared with 28 of 464 (6%) patients with CFS score <5 (log-rank *P* value <0.001). Of the 112 patients with SQ answer no, 19 died within 1 year (17%), compared with 33 of 477 (7%) patients with SQ answer yes (log-rank *P* value <0.001). Table 3 shows the results of the Cox proportional hazards model analyses for both CFS and SQ. Patients with CFS score ≥5 had a three-fold higher risk of death (fully adjusted hazard ratio [HR], 3.09; 95% CI, 1.75 to 5.54) and patients with SQ answer no had an almost two-fold significantly higher risk of death (fully adjusted HR, 1.96; 95% CI, 1.09 to 3.52).

**Table 3:** Hazard ratios for Clinical Frailty Scale, Surprise Question assessment, and 1-year all-cause mortality

Assessments	Model	Hazard Ratio	95% CI	*P* Value
CFS: ≥5 versus <5^a^	Model 1: crude	3.62	2.10 to 6.24	<0.001
	Model 2: partially adjusted	3.05	1.73 to 5.37	<0.001
	Model 3: fully adjusted	3.09	1.75 to 5.54	<0.001
SQ: no versus yes^b^	Model 1: crude model	2.51	1.43 to 4.42	0.001
	Model 2: partially adjusted	1.95	1.09 to 3.52	0.03
	Model 3: fully adjusted	1.96	1.09 to 3.52	0.03

Model 1 (crude): no adjustment for covariates.

Model 2 (partially adjusted): adjusted for age and sex.

Model 3 (fully adjusted): adjusted for age, sex and eGFR.

CFS, Clinical Frailty Scale; CI, confidence interval; SQ, Surprise Question.

a Clinical Frailty Scale score ≥5 considered high risk, *n*=125. Clinical Frailty Scale score <5 considered low risk, *n*=464 (reference group).

b Surprise Question answer no considered high risk, *n*=112. Surprise Question answer yes considered low risk, *n*=477 (reference group).

Group with Clinical Frailty Scale score ≥5 (high risk): 19% mortality rate after 1 year. Group with Clinical Frailty Scale score <5 (low risk) 6% mortality rate after 1 year. Group with Surprise Question answer no (high risk): 17% mortality rate after 1 year. Group with Surprise Question answer yes (low risk): 7% mortality rate after 1 year.

### Subgroups and 1-Year Mortality

**Figure 3:**
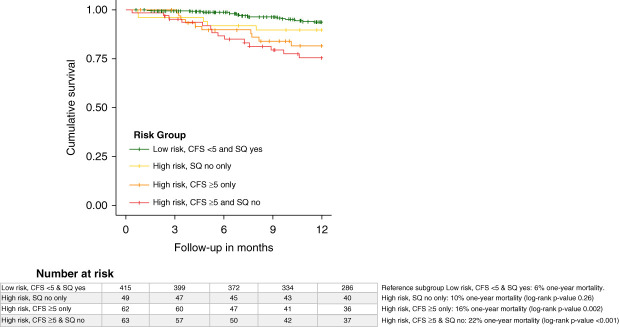
**Kaplan–Meier curve of the four CFS and SQ subgroups and 1-year mortality.** CFS, Clinical Frailty Scale; SQ, surprise question

Figure 3 shows the Kaplan–Meier curves and log-rank tests depicting 1-year mortality of the four subgroups. One-year mortality was 22%, 16%, 10%, and 6% for the subgroups high risk, CFS ≥5 & SQ no (*n*=63, 14 deaths); high risk, CFS ≥5 only (*n*=62, 10 deaths); high risk, SQ no only (*n*=43, 5 deaths); and low risk, CFS <5 & SQ yes (*N*=415, 23 deaths); respectively. In Table 4, the results from the Cox proportional hazards model analyses for the three subgroups are shown. Both subgroups high risk, CFS ≥5 & SQ no and high risk, CFS ≥5 only had significantly higher risk of death compared with the reference group (fully adjusted HR, 3.37; 95% CI, 1.65 to 6.91 and fully adjusted HR, 3.19; 95% CI, 1.51 to 6.76, respectively). No significant association with 1-year mortality was found in subgroup high risk, SQ no only (fully adjusted HR, 1.51; 95% CI, 0.56 to 4.06).

**Table 4:** Hazard ratios for Clinical Frailty Scale and Surprise Question subgroups and 1-year all-cause mortality versus reference group

Assessments	Model	Hazard Ratio	95% CI	*P* Value
High risk, CFS ≥5 & SQ no	Model 1: crude	4.53	2.34 to 8.84	<0.001
	Model 2: partially adjusted	3.34	1.64 to 6.83	<0.001
	Model 3: fully adjusted	3.37	1.65 to 6.91	<0.001
High risk, CFS ≥5 only	Model 1: crude	3.32	1.59 to 6.98	0.002
	Model 2: partially adjusted	3.12	1.47 to 6.61	0.003
	Model 3: fully adjusted	3.19	1.51 to 6.76	0.002
High risk, SQ no only	Model 1: crude	1.74	0.66 to 4.57	0.26
	Model 2: partially adjusted	1.51	0.56 to 4.06	0.42
	Model 3: fully adjusted	1.51	0.56 to 4.06	0.41

Clinical Frailty Scale ≥5 and Surprise Question answer no considered high risk, Clinical Frailty Scale <5 and Surprise Question answer yes considered low risk. Subgroup high risk, Clinical Frailty Scale ≥5 and Surprise Question no *n*=63. Subgroup high risk, Clinical Frailty Scale ≥5 only *n*=62. Subgroup High risk, Surprise Question no only *n*=49. Subgroup low risk, Clinical Frailty Scale<5 & Surprise Question yes (*N*=415) used as reference group.

Reference subgroup low risk, Clinical Frailty Scale<5 & Surprise Question yes 6% 1-year mortality. Subgroup high risk, Surprise Question no only 10% 1-year mortality. Subgroup high risk, Clinical Frailty Scale ≥5 only 16% 1-year mortality. Subgroup high risk, Clinical Frailty Scale ≥5 & Surprise Question no 22% mortality rate.

Model 1 (crude): no adjustments for covariates.

Model 2 (partially adjusted): adjusted for age and sex.

Model 3 (fully adjusted): adjusted for age, sex, and eGFR.

CFS, Clinical Frailty Scale; CI, confidence interval; SQ, Surprise Question.

## Discussion

This study shows that results from the CFS and SQ screening tools are significantly associated with 1-year mortality risk in older patients with advanced CKD. Combining results from both the CFS and SQ showed the strongest association with mortality compared with either tool individually. Thus, using these quick screening tools in conjunction may have potential added value for the estimation of early mortality risk in older patients with advanced CKD.

There is a need for nephrologists to identify older patients with high mortality risk who would probably not benefit from KRT. This risk identification can help to guide the SDM process regarding therapeutic options for kidney failure and facilitate timely initiation of conversations on ACP.^9,24^ As prognostic uncertainty is seen as a potential barrier for ACP by nephrologists, using easy and quick tools to identify patients with high mortality risk that are implementable by nephrology care providers may be helpful.^10,13,25^ Combining both is quick as well, and our findings suggest that combining quick frailty screening (using the CFS) with a clinician's more subjective clinical gestalt (SQ) may be of added value.

Previously, both frailty (assessed using various extensive tools) and the SQ individually have been shown to be significantly associated with mortality in different CKD populations.^6,13,22,26–28^ It should be noted, however, that these study populations mostly consisted of dialysis patients, patients with better preserved eGFR, or younger patients. To our knowledge, however, no study has previously explored both tools in the same population nor coupled them to explore potential associations with early mortality in older patients approaching kidney failure. We found the highest mortality risk in patients where both the CFS and SQ indicated high risk, which may be because of the complementary effect of combining more objective frailty screening with a subsequent assessment requiring subjective clinical gestalt to estimate the likelihood of death. Beyond likely taking the existing level of frailty into account (which may explain why the nonfrail subgroup high risk, SQ no only was the smallest), the SQ requires clinical impression based on (personal) experience to estimate the likelihood of further disease progression and prognosis.^12,14,29^ This additional consideration of prognosis, which is not necessary for frailty screening alone, may explain the higher mortality risk we found in patients where both assessments indicated high risk.

The results from this study have several potential clinical implications. Mortality risk assessment with the CFS and SQ is quick, has been implemented before by nephrology care providers, and can provide valuable information regarding early mortality risk in older patients approaching kidney failure.^10,13^ These tools thus may help to inform both nephrologists and their older patients with kidney failure when faced with the choice of either KRT or CKM or help to broach conversations regarding ACP.^8,23^ This may also be the case in nonfrail (*i.e*., CFS <5) patients with SQ answer no because we noted differences in baseline characteristics compared with nonfrail patients with SQ answer yes that may be clinically relevant (*e.g*., age, number of symptoms and medications, nutrition). Models such as KDpredict (which predicts both risk of death and risk of kidney failure) use readily available parameters (*e.g*., age, sex, eGFR, proteinuria, comorbidities) but do not incorporate quick and implementable frailty screening or subjective risk assessment (*i.e*., clinicians gestalt).^10,13,30^ Future research should explore whether adding the CFS and SQ (or a combination thereof) to existing mortality prediction models could improve individual mortality risk prediction in older patients with advanced CKD.

Frailty screening with the CFS in older patients approaching kidney failure may also be beneficial as it potentially allows for targeted interventions (*e.g*., increased exercise, dietary intervention) aimed at lessening frailty severity and improving clinical outcomes and HRQoL.^31^ A potential downside of using screening tools for frailty can be their poor discriminating ability to rule out frailty compared with comprehensive geriatric assessments in CKD populations, which may wrongfully classify patients as nonfrail.^32^ This may partly explain the low prevalence of frailty detected (21%) in our study, compared with a prevalence of approximately 35% found in an extensive review of 139 studies primary using more comprehensive frailty assessments, analyzing primarily KRT cohorts.^33^ The routine use of the gold standard for frailty assessment, which is a comprehensive geriatric assessments, is currently limited within nephrology care as it is resource demanding.^4^ Implementation of a nephrology-tailored geriatric assessment into routine care, which is less time consuming, could be a solution.^15,16,34^

Our study has various notable strengths. We explored associations between the results of both the CFS and SQ, individually and in combination, with 1-year mortality in a sizable cohort of older patients approaching kidney failure. Furthermore, our cohort represents a specific group of patients with CKD who are all age ≥65 years, with eGFR 20–10 ml/min per 1.73 m^2^, for whom early mortality risk identification may be particularly helpful when discussing KRT, CKM, and ACP. Finally, patients from 40 different hospitals in the Netherlands (38 hospitals) and Belgium (2 hospitals) were included in this study including nonacademic and academic hospitals, limiting potential center-specific effects.

Our study also has some limitations. First, both the CFS and SQ were often scored consecutively by the doctor or trained nurse or health care professional performing the nephrology-tailored assessment. This likely resulted in unavoidable anchoring bias, where the CFS assessment may have influenced the subsequent answering of the SQ.^35^ Thus, the interpretative independence of the association between the SQ and mortality may be limited. It should be noted, however, that within patients with CFS score ≥5, a higher mortality risk was observed when SQ answer was no compared with yes, indicating some independent added value of assessing the SQ after the CFS despite potential anchoring bias. Second, selection bias may have affected our findings as patients who were excluded because of lacking CFS and/or SQ assessment differed significantly in characteristics associated with frailty and mortality risk (*e.g*., age, ADL-Katz scores) and had a significantly higher mortality risk as well. This likely resulted in an underestimation of the associations between assessments and early mortality, as a particularly frail group was likely excluded. Despite this, however, strong associations were found with mortality even without this excluded group, highlighting the potential value of these screening tools. Third, no data were gathered regarding the race or ethnicity of our study population. This may affect the potential generalizability of our findings because frailty may differ across ethnicities.^36^ Furthermore, both the Netherlands and Belgium have universal health care with almost 100% health care insurance coverage, making it unclear whether our findings are generalizable to countries with different nephrology practices and fewer resources.^37^ Finally, our results reflect associations between CFS and SQ assessment groups and mortality, but not individual patient mortality risk. Future research using predictive models should be done to assess the usefulness of both tools and their combination for individual risk prediction.

In conclusion, both the results of the CFS and SQ are significantly associated with 1-year mortality in older patients with advanced CKD. Combining both assessments may have added value as the strongest association with mortality risk is found using their combination when both indicate high risk, compared to using either tool individually. Both assessments are easy to implement into standard nephrology care, and may help (*1*) identify patients who are vulnerable, (*2*) assist older patients and nephrologists in making treatment decisions between KRT and CKM, and (*3*) help with timely initiation of ACP.

## Supplementary Material

## Data Availability

Original data generated for the study will be made available upon reasonable request to the corresponding author. Observational Data. The data that support the findings of this study are available from the corresponding author upon reasonable request.
