# Supplementary material for: Clinical Frailty Scale, Surprise Question and 1-year Mortality in Older Patients with Advanced CKD
Source: Kidney360. 2025 Aug 21;7(1):107–16. doi: 10.34067/KID.0000000936 (PMC12889955; doi:10.34067/KID.0000000936)
Supplement: Supplementary file 1 [file kidney360-7-107-s001.pdf]

ASN American Society of Nephrology | JASN Journal of the American Society of Nephrology | KIDNEY360 Accessing Our World From Every Angle | CJASN Clinical Journal of the American Society of Nephrology

ASN Journal Disclosure Form

As per ASN journal policy, I have disclosed any financial relationships or commitments I have held in the past 36 months as included below. I have listed my Current Employer below to indicate there is a relationship requiring disclosure. If no relationship exists, my Current Employer is not listed.

A. Abrahams reports the following:
Employer: University Medical Center Utrecht; and Research Funding: Baxter; Fresenius Medical Care; Dutch Kidney Foundation; Astellas.

I understand that the information above will be published within the journal article, if accepted, and that failure to comply and/or to accurately and completely report the potential financial conflicts of interest could lead to the following: 1) Prior to publication, article rejection, or 2) Post-publication, sanctions ranging from, but not limited to, issuing a correction, reporting the inaccurate information to the authors' institution, banning authors from submitting work to ASN journals for varying lengths of time, and/or retraction of the published work.

Name: Alferso C. Abrahams
Manuscript ID: K360-2025-000550R1
Manuscript Title: Clinical Frailty Scale, Surprise Question and one-year mortality in older patients with advanced CKD
Date of Completion: July 24, 2025
Disclosure Updated Date: September 12, 2024

ASN Journal Disclosure Form

As per ASN journal policy, I have disclosed any financial relationships or commitments I have held in the past 36 months as included below. I have listed my Current Employer below to indicate there is a relationship requiring disclosure. If no relationship exists, my Current Employer is not listed.

W. Bos reports the following:
Employer: St. Antonius Hospital; Leiden University Medical Center; Research Funding: Dutch Kidney Foundation; ZonMW (The Netherlands Organisation for Health Research and Development); St Antonius Research Fund; and Advisory or Leadership Role: chair ICHOM working group CKD; committee-member of the Dutch Nephrology Quality Initiative "Nefrovisie", co-chair of Linnean Initiative.

I understand that the information above will be published within the journal article, if accepted, and that failure to comply and/or to accurately and completely report the potential financial conflicts of interest could lead to the following: 1) Prior to publication, article rejection, or 2) Post-publication, sanctions ranging from, but not limited to, issuing a correction, reporting the inaccurate information to the authors' institution, banning authors from submitting work to ASN journals for varying lengths of time, and/or retraction of the published work.

Name: Willem Jan W. Bos
Manuscript ID: K360-2025-000550R1
Manuscript Title: Clinical Frailty Scale, Surprise Question and one-year mortality in older patients with advanced CKD,
Date of Completion: July 24, 2025
Disclosure Updated Date: July 24, 2025

ASN
American Society of Nephrology
JASN
Journal of the American Society of Nephrology
KIDNEY360
Accessing Our World From Every Angle
CJASN
Clinical Journal of the American Society of Nephrology

ASN Journal Disclosure Form

As per ASN journal policy, I have disclosed any financial relationships or commitments I have held in the past 36 months as included below. I have listed my Current Employer below to indicate there is a relationship requiring disclosure. If no relationship exists, my Current Employer is not listed.

T. Cnossen reports the following:
Employer: Amphia Hospital

I understand that the information above will be published within the journal article, if accepted, and that failure to comply and/or to accurately and completely report the potential financial conflicts of interest could lead to the following: 1) Prior to publication, article rejection, or 2) Post-publication, sanctions ranging from, but not limited to, issuing a correction, reporting the inaccurate information to the authors' institution, banning authors from submitting work to ASN journals for varying lengths of time, and/or retraction of the published work.

Name: Trijntje T. Cnossen
Manuscript ID: K360-2025-000550R1
Manuscript Title: Clinical Frailty Scale, Surprise Question and one-year mortality in older patients with advanced CKD
Date of Completion: July 28, 2025
Disclosure Updated Date: July 28, 2025

ASN
American Society of Nephrology
JASN
Journal of the American Society of Nephrology
KIDNEY360
Accessing Our World From Every Angle
CJASN
Clinical Journal of the American Society of Nephrology

ASN Journal Disclosure Form

As per ASN journal policy, I have disclosed any financial relationships or commitments I have held in the past 36 months as included below. I have listed my Current Employer below to indicate there is a relationship requiring disclosure. If no relationship exists, my Current Employer is not listed.

I. Demirhan reports the following:
Employer: University Medical Center Utrecht & Leiden University Medical Center

I understand that the information above will be published within the journal article, if accepted, and that failure to comply and/or to accurately and completely report the potential financial conflicts of interest could lead to the following: 1) Prior to publication, article rejection, or 2) Post-publication, sanctions ranging from, but not limited to, issuing a correction, reporting the inaccurate information to the authors' institution, banning authors from submitting work to ASN journals for varying lengths of time, and/or retraction of the published work.

Name: Imre Demirhan
Manuscript ID: K360-2025-000550R1
Manuscript Title: Clinical Frailty Scale, Surprise Question and one-year mortality in older patients with advanced CKD
Date of Completion: July 25, 2025
Disclosure Updated Date: July 25, 2025

ASN American Society of Nephrology JASN Journal of the American Society of Nephrology KIDNEY360 Accessing Our World From Every Angle CJASN Clinical Journal of the American Society of Nephrology

ASN Journal Disclosure Form

As per ASN journal policy, I have disclosed any financial relationships or commitments I have held in the past 36 months as included below. I have listed my Current Employer below to indicate there is a relationship requiring disclosure. If no relationship exists, my Current Employer is not listed.

M. Jongejan has nothing to disclose.

I understand that the information above will be published within the journal article, if accepted, and that failure to comply and/or to accurately and completely report the potential financial conflicts of interest could lead to the following: 1) Prior to publication, article rejection, or 2) Post-publication, sanctions ranging from, but not limited to, issuing a correction, reporting the inaccurate information to the authors' institution, banning authors from submitting work to ASN journals for varying lengths of time, and/or retraction of the published work.

Name: Micha Jongejan
Manuscript ID: K360-2025-000550R1
Manuscript Title: Clinical Frailty Scale, Surprise Question and one-year mortality in older patients with advanced CKD
Date of Completion: July 18, 2025
Disclosure Updated Date: July 18, 2025

ASN American Society of Nephrology JASN Journal of the American Society of Nephrology KIDNEY360 Accessing Our World From Every Angle CJASN Clinical Journal of the American Society of Nephrology

ASN Journal Disclosure Form

As per ASN journal policy, I have disclosed any financial relationships or commitments I have held in the past 36 months as included below. I have listed my Current Employer below to indicate there is a relationship requiring disclosure. If no relationship exists, my Current Employer is not listed.

J. Joosten reports the following:
Employer: MUMC- Maastricht University Medical Centre; and Advisory or Leadership Role: Board member of the subdivision Geriatric medicine of the Dutch Internal Medicine Association (NIV).

I understand that the information above will be published within the journal article, if accepted, and that failure to comply and/or to accurately and completely report the potential financial conflicts of interest could lead to the following: 1) Prior to publication, article rejection, or 2) Post-publication, sanctions ranging from, but not limited to, issuing a correction, reporting the inaccurate information to the authors' institution, banning authors from submitting work to ASN journals for varying lengths of time, and/or retraction of the published work.

Name: Jmh Joosten
Manuscript ID: K360-2025-000550R1
Manuscript Title: Clinical Frailty Scale, Surprise Question and one-year mortality in older patients with advanced CKD
Date of Completion: July 28, 2025
Disclosure Updated Date: July 28, 2025

ASN
American Society of Nephrology
JASN
Journal of the American Society of Nephrology
KIDNEY360
Accessing Our World From Every Angle
CJASN
Clinical Journal of the American Society of Nephrology

ASN Journal Disclosure Form

As per ASN journal policy, I have disclosed any financial relationships or commitments I have held in the past 36 months as included below. I have listed my Current Employer below to indicate there is a relationship requiring disclosure. If no relationship exists, my Current Employer is not listed.

K. Kiriwenno reports the following:
Employer: University Medical Center Utrecht

I understand that the information above will be published within the journal article, if accepted, and that failure to comply and/or to accurately and completely report the potential financial conflicts of interest could lead to the following: 1) Prior to publication, article rejection, or 2) Post-publication, sanctions ranging from, but not limited to, issuing a correction, reporting the inaccurate information to the authors' institution, banning authors from submitting work to ASN journals for varying lengths of time, and/or retraction of the published work.

Name: Keanu B. Kiriwenno
Manuscript ID: K360-2025-000550R1
Manuscript Title: Clinical Frailty Scale, Surprise Question and one-year mortality in older patients with advanced CKD
Date of Completion: July 21, 2025
Disclosure Updated Date: July 21, 2025

ASN American Society of Nephrology | JASN Journal of the American Society of Nephrology | KIDNEY360 Accessing Our World From Every Angle | CJASN Clinical Journal of the American Society of Nephrology

ASN Journal Disclosure Form

As per ASN journal policy, I have disclosed any financial relationships or commitments I have held in the past 36 months as included below. I have listed my Current Employer below to indicate there is a relationship requiring disclosure. If no relationship exists, my Current Employer is not listed.

S. Mooijaart reports the following:
Employer: Leiden University Medical Center

I understand that the information above will be published within the journal article, if accepted, and that failure to comply and/or to accurately and completely report the potential financial conflicts of interest could lead to the following: 1) Prior to publication, article rejection, or 2) Post-publication, sanctions ranging from, but not limited to, issuing a correction, reporting the inaccurate information to the authors' institution, banning authors from submitting work to ASN journals for varying lengths of time, and/or retraction of the published work.

Name: Simon Mooijaart
Manuscript ID: K360-2025-000550R1
Manuscript Title: Clinical Frailty Scale, Surprise Question and one-year mortality in older patients with advanced CKD
Date of Completion: July 18, 2025
Disclosure Updated Date: July 18, 2025

ASN American Society of Nephrology · JASN Journal of the American Society of Nephrology · KIDNEY360 Accessing Our World From Every Angle · CJASN Clinical Journal of the American Society of Nephrology

ASN Journal Disclosure Form

As per ASN journal policy, I have disclosed any financial relationships or commitments I have held in the past 36 months as included below. I have listed my Current Employer below to indicate there is a relationship requiring disclosure. If no relationship exists, my Current Employer is not listed.

M. Van Buren reports the following:
Research Funding: Astra Zeneca; Boehringer Ingelheim; and Advisory or Leadership Role: Vifor Pharma.

I understand that the information above will be published within the journal article, if accepted, and that failure to comply and/or to accurately and completely report the potential financial conflicts of interest could lead to the following: 1) Prior to publication, article rejection, or 2) Post-publication, sanctions ranging from, but not limited to, issuing a correction, reporting the inaccurate information to the authors' institution, banning authors from submitting work to ASN journals for varying lengths of time, and/or retraction of the published work.

Name: Marjolyn Van Buren
Manuscript ID: K360-2025-000550R1
Manuscript Title: Clinical Frailty Scale, Surprise Question and one-year mortality in older patients with advanced CKD
Date of Completion: July 21, 2025
Disclosure Updated Date: July 21, 2025

ASN American Society of Nephrology | JASN Journal of the American Society of Nephrology | KIDNEY360 Accessing Our World From Every Angle | CJASN Clinical Journal of the American Society of Nephrology

ASN Journal Disclosure Form

As per ASN journal policy, I have disclosed any financial relationships or commitments I have held in the past 36 months as included below. I have listed my Current Employer below to indicate there is a relationship requiring disclosure. If no relationship exists, my Current Employer is not listed.

M. Van Oevelen reports the following:
Employer: Leiden University Medical Center

I understand that the information above will be published within the journal article, if accepted, and that failure to comply and/or to accurately and completely report the potential financial conflicts of interest could lead to the following: 1) Prior to publication, article rejection, or 2) Post-publication, sanctions ranging from, but not limited to, issuing a correction, reporting the inaccurate information to the authors' institution, banning authors from submitting work to ASN journals for varying lengths of time, and/or retraction of the published work.

Name: Mathijs Van Oevelen
Manuscript ID: K360-2025-000550R1
Manuscript Title: Clinical Frailty Scale, Surprise Question and one-year mortality in older patients with advanced CKD
Date of Completion: July 21, 2025
Disclosure Updated Date: July 21, 2025

ASN American Society of Nephrology | JASN Journal of the American Society of Nephrology | KIDNEY360 Accessing Our World From Every Angle | CJASN Clinical Journal of the American Society of Nephrology

ASN Journal Disclosure Form

As per ASN journal policy, I have disclosed any financial relationships or commitments I have held in the past 36 months as included below. I have listed my Current Employer below to indicate there is a relationship requiring disclosure. If no relationship exists, my Current Employer is not listed.

M. Verhaar reports the following:
Advisory or Leadership Role: Scientific Advisory Board ERA; Scientific Board Dutch Kidney Foundation

I understand that the information above will be published within the journal article, if accepted, and that failure to comply and/or to accurately and completely report the potential financial conflicts of interest could lead to the following: 1) Prior to publication, article rejection, or 2) Post-publication, sanctions ranging from, but not limited to, issuing a correction, reporting the inaccurate information to the authors' institution, banning authors from submitting work to ASN journals for varying lengths of time, and/or retraction of the published work.

Name: Marianne C. Verhaar
Manuscript ID: K360-2025-000550R1
Manuscript Title: Clinical Frailty Scale, Surprise Question and one-year mortality in older patients with advanced CKD
Date of Completion: July 18, 2025
Disclosure Updated Date: July 18, 2025
