# Supplementary material for: Clinical Frailty Scale, Surprise Question and 1-year Mortality in Older Patients with Advanced CKD
Source: Kidney360. 2025 Aug 21;7(1):107–16. doi: 10.34067/KID.0000000936 (PMC12889955; doi:10.34067/KID.0000000936)
Supplement: Supplementary file 2 [file kidney360-7-107-s002.pdf]

SUPPLEMENTAL MATERIAL

Supplemental figure 1: Flowchart showing study participant selection

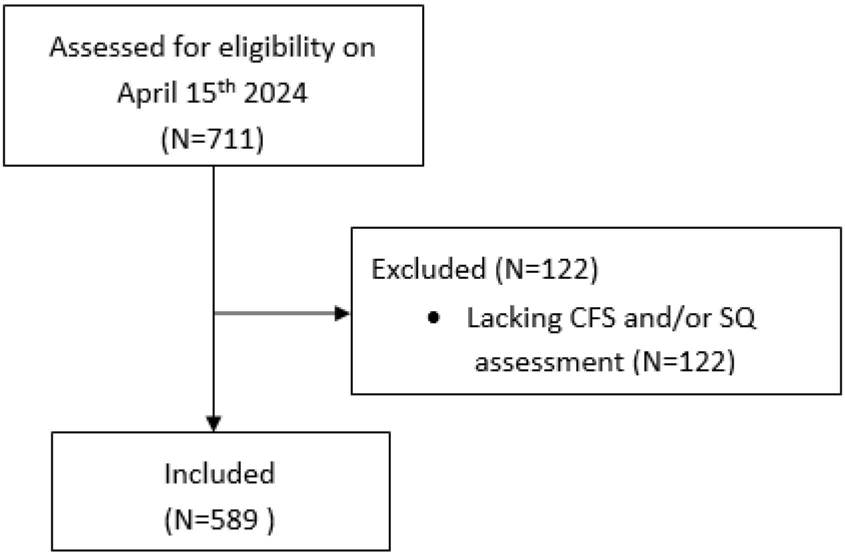

Supplemental Table 1: Baseline patient characteristics of included and excluded patients

Characteristics	Included patients (N=589)	Excluded patients (N=122)	P-value
Age in yr, mean (SD)	77 (6)	78 (6)	0.02
Male sex, *No.* (%)	410 (70)	81 (66)	0.48
eGFR in ml/ min per 1.73 m^2, mean (SD)	15 (3)	15 (3)	0.69
**Primary kidney disease, *No.* (%)**			0.94
Diabetic kidney disease	123 (21)	24 (23)	
GN	30 (5)	6 (6)	
Hypertension	107 (19)	22 (21)	
Cystic kidney disease	25 (4)	3 (3)	
Pyelonephritis	4 (1)	0 (0)	
Renal vascular disease	105 (18)	15 (15)	
Other	149 (26)	27 (26)	
Unknown	34 (6)	6 (6)	
**Living situation, *No.* (%)**			0.46
Alone, independently	200 (38)	33 (41)	
With partner	302 (58)	43 (54)	
With family	17 (3)	2 (3)	
Nursing home	4 (1)	2 (3)	
**Educational level, *No.* (%)**[a]			0.04
Low	198 (37)	41 (51)	
Medium	181 (34)	23 (28)	
High	161 (30)	17 (21)	
ADL-Katz score, median (IQR)[b]	0 (0 – 1)	1 (0 – 1)	0.03
IADL-Lawton score, median (IQR)[b]	5 (5 – 5)	5 (4 – 5)	0.15
No. of DSI symptoms, median (IQR)	10 (6 – 14)	12 (9 – 17)	0.005
Charlson Comorbidity Index score, median (IQR)	4 (3 – 5)	5 (3 – 6)	0.27
No. of medications, median (IQR)	11 (8 – 14)	11 (9 – 13)	0.14
**Mini nutritional assessment status, *No.* (%)**			0.41
Well nourished	429 (74)	21 (70)	
At risk of malnutrition	136 (23)	7 (23)	
Malnourished	15 (3)	2 (7)	
Serum albumin in g/L, mean (SD)	38.3 (5.3)	38.1 (6.7)	0.75
**Smoking status, *No.* (%)**			0.31
Quit	346 (63)	55 (70)	

Current smoker	46 (8)	3 (4)	
**Alcohol use, *No.* (%)**			0.22
Never or previously quit	310 (58)	45 (56)	
Current user	228 (42)	35 (44)	

Differences assessed using T-tests, Chi-Square tests and Mann-Whitney U tests. eGFR, estimated glomerular filtration rate; GN, glomerulonephritis; ADL, Activities of Daily Living; IADL, Instrumental Activities of Daily Living; DSI, Dialysis Symptom Index.

[a]Based on the Dutch Verhage education classification. [b]ADL: Activities of Daily Living, basic and essential self-care tasks. Lower ADL-Katz score reflects more independence, range 0 - 12. IADL: Instrumental Activities of Daily Living, more complex ADL tasks. Higher IADL-Lawton score reflects more independence, range 0 – 5 in men and 0 – 8 in women. Missing values in the included patients: Serum albumin 14%, Primary Kidney Disease 2%, living situation 11%, educational level 8%, ADL-Katz score 13%, IADL-Lawton score 12%, number of DSI symptoms 6%, number of medications 2%, Mini Nutritional Assessment 2%, smoking status 7%, alcohol use 9%. Missing values in the excluded patients: Primary Kidney Disease 16%, living situation 34%, educational level 34%, ADL-Katz score 33%, IADL-Lawton score 33%, number of DSI symptoms 34%, Charlson Comorbidity Index score 74%, number of medications 16%, Mini Nutritional Assessment 75%, serum albumin 26%, smoking status 35%, alcohol use 34%.

Supplemental material: Filled in STROBE checklist

	Item No.	Recommendation	Page No.	Relevant text from manuscript
**Title and abstract**	1	(*a*) Indicate the study's design with a commonly used term in the title or the abstract	1	See abstract: "…were included from the ongoing prospective observational cohort study DIALysis or not: Outcomes in older kidney patients with GerIatriC Assessment (first inclusion May 13th, 2020)."
		(*b*) Provide in the abstract an informative and balanced summary of what was done and what was found	1	See abstract: Sections 'Methods', 'Results' and 'Conclusion'
**Introduction**				
Background/rationale	2	Explain the scientific background and rationale for the investigation being reported	1,2	"It remains unclear whether frailty and risk assessment using the CFS and SQ are associated with early mortality in specifically older patients with advanced CKD approaching kidney failure and whether combining both is associated with higher mortality risk. Exploring this may provide important information to guide SDM for nephrologists and their patients."
Objectives	3	State specific objectives, including any prespecified hypotheses	2	"Thus, this study aims to investigate the association between both frailty and clinical impression using the CFS and SQ with one-year mortality in older patients with advanced CKD. Furthermore, this study explores whether combining both assessments is of added value compared with either assessment individually."
**Methods**				
Study design	4	Present key elements of study design early in the paper	2	See 'Study design and participants': "This study included patients from the DIALysis or not: Outcomes in older kidney patients with GerIatriC Assessment (DIALOGICA) study, which is an ongoing multicenter prospective observational study in 40 hospitals in the Netherlands (38 hospitals) and Belgium (2 hospitals)."
Setting	5	Describe the setting, locations, and relevant dates, including periods of recruitment, exposure, follow-up, and data collection	2,3	See 'study design and participants', and 'Outcome assessment'
Participants	6	(*a*) *Cohort study*—Give the eligibility criteria, and the sources and methods	2,3	See 'Study design and participants', and

		of selection of participants. Describe methods of follow-up<br><br>*Case-control study*—Give the eligibility criteria, and the sources and methods of case ascertainment and control selection. Give the rationale for the choice of cases and controls<br><br>*Cross-sectional study*—Give the eligibility criteria, and the sources and methods of selection of participants		'Outcome assessment'
		(*b*) *Cohort study*—For matched studies, give matching criteria and number of exposed and unexposed<br><br>*Case-control study*—For matched studies, give matching criteria and the number of controls per case		N.A.
Variables	7	Clearly define all outcomes, exposures, predictors, potential confounders, and effect modifiers. Give diagnostic criteria, if applicable	2,3	See sections 'Clinical Frailty Scale and Surprise Question', 'Outcome assessment' and 'Statistical analyses'.
Data sources/ measurement	8*	For each variable of interest, give sources of data and details of methods of assessment (measurement). Describe comparability of assessment methods if there is more than one group	2,3	See section 'Study design and participants' and 'Measurements'.
Bias	9	Describe any efforts to address potential sources of bias	3	See 'Statistical analyses'<br><br>"The Cox proportional hazards models were adjusted for potential confounders, which were added in steps. Model 1 comprised only either the CFS or SQ assessment (crude), model 2 added age and sex (partially adjusted), and model 3 finally added eGFR (fully adjusted).<br><br>"To explore whether selection bias may have affected our analyses, the 1-year mortality risk of included and excluded patients (lacking CFS and/or SQ assessment) were compared as well by using a Cox proportional hazards model."
Study size	10	Explain how the study size was arrived at	2	See 'Study design and participants'.<br><br>"For this study, all DIALOGICA participants who were included from May 13th, 2020, up to April 15th, 2024, who had data available on both the CFS

				and SQ assessments at baseline were included.”

Continued on next page

Quantitative variables	11	Explain how quantitative variables were handled in the analyses. If applicable, describe which groupings were chosen and why		See ‘Measurements’
Statistical methods	12	(*a*) Describe all statistical methods, including those used to control for confounding	3	See ‘Statistical analyses’
		(*b*) Describe any methods used to examine subgroups and interactions	3	See ‘Statistical analyses’
		(*c*) Explain how missing data were addressed	3	“None of the covariates used in the multivariable models had missing values.”
		(*d*) *Cohort study*—If applicable, explain how loss to follow-up was addressed<br>*Case-control study*—If applicable, explain how matching of cases and controls was addressed<br>*Cross-sectional study*—If applicable, describe analytical methods taking account of sampling strategy	3	See ‘Outcome assessment’<br>“Patient survival was censored when consent was withdrawn by the patient, in the case of patient transfer to a different nephrology care facility not participating in DIALOGICA (loss to follow-up) or in the case of follow-up time <1 year (patients included <12 months before April 15th, 2024).”
		(*e*) Describe any sensitivity analyses		N.A.
**Results**				
Participants	13*	(a) Report numbers of individuals at each stage of study—eg numbers potentially eligible, examined for eligibility, confirmed eligible, included in the study, completing follow-up, and analysed	3	“On April 15th, 2024, 711 patients were included in the DIALOGICA study. In total, 589 patients of these patients (83%) had both CFS and SQ assessments available and were thus included in this study (displayed in Supplemental Figure 1).”<br>“Of the 589 patients at baseline, 15 (3%) were lost to follow-up, and 52 patients (9%) had died within 1 year.”
		(b) Give reasons for non-participation at each stage	3	“…15 (3%) were lost to follow up,…”
		(c) Consider use of a flow diagram		See supplemental figure 1.
Descriptive data	14*	(a) Give characteristics of study participants (eg demographic, clinical, social) and	4,6	See tables 1 and 2

		information on exposures and potential confounders		
		(b) Indicate number of participants with missing data for each variable of interest	4	See table 1. “Missing values: Serum albumin 14%, Primary Kidney Disease 2%, living situation 11%, educational level 8.3%, ADL-Katz score 13%, IADL-Lawton score 12%, number of DSI symptoms 6%, number of medications 2%, Mini Nutritional Assessment 2%, smoking status 7%, alcohol use 9%.”
		(c) *Cohort study*—Summarise follow-up time (eg, average and total amount)	3	“The median follow-up time was 12 months (IQR 10 – 12)”
Outcome data	15*	*Cohort study*—Report numbers of outcome events or summary measures over time	3	“Of the 589 patients at baseline, 15 (3%) were lost to follow up, and 52 patients (9%) had died within one year.”
		Case-control study—Report numbers in each exposure category, or summary measures of exposure		N.A.
		Cross-sectional study—Report numbers of outcome events or summary measures		N.A.
Main results	16	(*a*) Give unadjusted estimates and, if applicable, confounder-adjusted estimates and their precision (eg, 95% confidence interval). Make clear which confounders were adjusted for and why they were included	3, 7, 8	See ‘Results’ and tables 3 and 4.
		(*b*) Report category boundaries when continuous variables were categorized	2	“CFS scores of $5 and ,5 were used to group patients as frail (thus high risk) and nonfrail (low risk), respectively, in line with the original and most frequently used cutoff point.” CFS-scores of ≥5 and <5 were used to group patients as frail (thus ‘high risk’) and non-frail (‘low risk’) respectively, in line with the original and most frequently used cut-off point.”
		(*c*) If relevant, consider translating estimates of relative risk into absolute risk for a meaningful time period		N.A.

Continued on next page

Other analyses	17	Report other analyses done—eg analyses of subgroups and interactions, and sensitivity analyses	2,3	"Patients were categorized into four risk subgroups based on the results of the CFS en SQ assessments: High risk: CFS <5 & SQ no; high risk: CFS <5 only; high risk: SQ no only; and low risk: CFS <5 & SQ yes (used as reference group for subgroup analyses)."<br><br>"To assess whether combining the results of both the CFS and SQ is of added value for mortality risk estimation, associations between the four subgroups and 1-year mortality were explored as well using the same steps, using the subgroup low risk: CFS >5 & SQ yes as the reference group.<br><br>"To explore whether selection bias may have affected our analyses, the 1-year mortality risk of included and excluded patients (lacking CFS and/or SQ assessment) were compared as well by using a Cox proportional hazards model."<br><br>Also see table 4.
**Discussion**				
Key results	18	Summarise key results with reference to study objectives	3,4	See first paragraph of 'Discussion'
Limitations	19	Discuss limitations of the study, taking into account sources of potential bias or imprecision. Discuss both direction and magnitude of any potential bias	6,7	See 'Strengths and limitations'
Interpretation	20	Give a cautious overall interpretation of results considering objectives, limitations, multiplicity of analyses, results from similar studies, and other relevant evidence	3-8	See 'discussion'
Generalisability	21	Discuss the generalisability (external validity) of the study results	7,8	"Thirdly, no data was gathered regarding the race or ethnicity of our study population. This may affect the potential generalizability of our findings, as frailty may differ across ethnicities. Furthermore, both the Netherlands and Belgium have universal health care with almost 100% healthcare insurance coverage, making it unclear whether our findings are generalizable to countries with different nephrology practices and fewer resources."

Other information				
Funding	22	Give the source of funding and the role of the funders for the present study and, if applicable, for the original study on which the present article is based	9	See ‘Funding’ and ‘Acknowledgements’

*Give information separately for cases and controls in case-control studies and, if applicable, for exposed and unexposed groups in cohort and cross-sectional studies.

Note: An Explanation and Elaboration article discusses each checklist item and gives methodological background and published examples of transparent reporting. The STROBE checklist is best used in conjunction with this article (freely available on the Web sites of PLoS Medicine at http://www.plosmedicine.org/, Annals of Internal Medicine at http://www.annals.org/, and Epidemiology at http://www.epidem.com/). Information on the STROBE Initiative is available at www.strobe-statement.org.
